# Splenomegaly in myelofibrosis—new options for therapy and the therapeutic potential of Janus kinase 2 inhibitors

**DOI:** 10.1186/1756-8722-5-43

**Published:** 2012-08-01

**Authors:** Jasleen Randhawa, Alen Ostojic, Radovan Vrhovac, Ehab Atallah, Srdan Verstovsek

**Affiliations:** 1 Medical College of Wisconsin, Milwaukee, WI, USA; 2 Division of Hematology, Department of Internal Medicine, University Hospital Center Zagreb, Zagreb, Croatia; 3 University of Zagreb School of Medicine, Zagreb, Croatia; 4 Department of Leukemia, University of Texas MD Anderson Cancer Center, Houston, TX, USA

**Keywords:** JAK2 inhibitor, Myeloproliferative neoplasms, Myelofibrosis, JAK2V617F mutation, Splenomegaly

## Abstract

Splenomegaly is a common sign of primary myelofibrosis (PMF), post-polycythemia vera myelofibrosis (post-PV MF), and post-essential thrombocythemia myelofibrosis (post-ET MF) that is associated with bothersome symptoms, which have a significant negative impact on patients’ quality of life. It may also be present in patients with advanced polycythemia vera (PV) or essential thrombocythemia (ET). Until recently, none of the therapies used to treat MF were particularly effective in reducing splenomegaly. The discovery of an activating Janus kinase 2 (JAK2) activating mutation (JAK2V617F) that is present in almost all patients with PV and in about 50-60 % of patients with ET and PMF led to the initiation of several trials investigating the clinical effectiveness of various JAK2 (or JAK1/JAK2) inhibitors for the treatment of patients with ET, PV, and MF. Some of these trials have documented significant clinical benefit of JAK inhibitors, particularly in terms of regression of splenomegaly. In November 2011, the US Food and Drug Administration approved the use of the JAK1- and JAK2-selective inhibitor ruxolitinib for the treatment of patients with intermediate or high-risk myelofibrosis, including PMF, post-PV MF, and post-ET MF. This review discusses current therapeutic options for splenomegaly associated with primary or secondary MF and the treatment potential of the JAK inhibitors in this setting.

## Introduction

Myelofibrosis (MF) is a clonal stem cell malignancy that presents clinically with 3 cardinal problems: progressive anemia, splenomegaly, and chronic incapacitating symptoms such as fatigue, bone pain, fever, night sweats, and weight loss [1]. It is one of 3 Philadelphia chromosome–negative myeloproliferative neoplasms (MPNs) that share elements of pathogenesis and symptomatology that may be related to dysregulated Janus kinase (JAK) signaling [2-4]. The other two are polycythemia vera (PV) and essential thrombocythemia (ET).

Splenomegaly, resulting from extramedullary hematopoiesis, accounts for some of the most debilitating symptoms of MF, whether primary or secondary to PV or ET. It contributes to the morbidity associated with MF by causing early satiety, dysregulated gastrointestinal function, portal hypertension, decreased physical activity, distressing abdominal pain, and worsening of assorted cytopenias secondary to splenic sequestration [5]. About 10 % of MF patients present with severely symptomatic splenomegaly at diagnosis; another 50 % will develop it within 4 years [6].

The relationship between dysregulated JAK signaling and the signs and symptoms of MPNs is well established [2-4]. The understanding of dysregulated JAK-STAT (signal transducer and activator of transcription) activity in MPNs as the basic pathophysiologic abnormality in practically all patients with MPNs has led to the clinical development of several JAK2 inhibitors. This review will discuss the therapies (apart from bone marrow transplant) currently used to treat splenomegaly and splenomegaly-related symptoms in patients with MF and the potential role of the JAK2 (as well as JAK1/JAK2) inhibitors.

## Current therapies

### Surgical therapy

Splenectomy has a long history of providing palliative relief for select patients with symptomatic splenomegaly. Although surgical technique and the availability of specific surgical expertise have improved, the procedure remains prone to significant rates of peri- and post-operative morbidity and mortality. Results of a retrospective study of 314 patients with MF-related splenomegaly who underwent splenectomy at the Mayo Clinic-Rochester between 1976 and 2004 show a perioperative complication rate of 27.7 % and a mortality rate due to surgical complications of 6.7 % [5]. The median OS after splenectomy was 19 months with 48 %, 50 %, and 40 % of patients experiencing an improvement in splenomegaly-related symptoms, anemia, and portal hypertension, respectively. No difference in post-splenectomy OS was noted when analyzed against leukemic transformation, MF risk score, decade during which splenectomy was performed, or type of MF.

Patient selection is critical to limiting peri- and post-operative morbidity. Preoperative thrombocytopenia with platelets < 50x10^9^/L appears to be associated with significantly worse OS. Patients with a history or suspicion of splenic infarct may be prone to a higher incidence of postoperative bleeding [5]. Splenectomy is appropriate only for patients with substantial splenic symptoms unresponsive to at least 1 prior medical therapy and those with an adequate performance status and a life expectancy of more than 1 year. Patients should be in otherwise good health without decompensated coagulopathy or significant comorbidities.

### Radiotherapy

Splenic irradiation is used in select patients to control various debilitating MF-associated symptoms. In general, the most appropriate candidates are those with significant symptoms and an adequate platelet count who, because of age or comorbidities, are not candidates for splenectomy [7]. Response rates ranging from 63 % to 95 % have been reported [8,9]. The results are transient, however, lasting for a median of only 6 months in one study [8] and 3.5 months in another [9]. The complications, which include myelosuppression, can be severe or even life-threatening. Significant side effects include potentially critical cytopenias, hemorrhage, complicated post-irradiation splenectomy [8] and delayed hemorrhage [7].

Recently, 2 cases were reported in which a regimen of induction-maintenance radiation therapy was used to treat MF with a marked improvement of the underlying accelerated phase of the disease [10]. At induction doses of 100 cGy (in 4 fractions of 25 cGy/fraction) with maintenance at the same or higher level, there was complete resolution of peripheral leukoerythroblastosis and eradication of peripheral blasts in one patient, and significant reduction in leukoerythroblastosis in the other. Both patients had marked improvement in functional status and a reduction in spleen size from >30 cm below the left costal margin (LCM) to 22 cm and 15 cm. The authors of this report concluded that this regimen was well tolerated and should be considered in specific clinical situations (e.g., rapid growth of spleen).

### Medical therapy

Prior to the FDA’s approval of ruxolitinib, medical therapies used to treat MF in clinical practice were limited to hydroxyurea, immunomodulatory drugs, alkylating agents, hypomethylating agents, and other chemotherapeutic agents (e.g., cladribine). A comparison of existing and investigational medical therapies is presented in Table 1.

**Table 1:** Comparison of Selected Drugs in Terms of a Response

**Agent**	**Splenomegaly**^**1**^	**Anemia**^**2**^	**Symptoms**^**3**^	**References**
**IMiDs**				
** Thalidomide**	19 %	43 %	NS	[15]
(Celgene Corp., Summit, NJ, USA)	8 %	22 %	NR	[16]
** Lenalidomide**	10 %	19 %	NR	[48]
(Celgene Corp., Summit, NJ, USA)	42 %	30 %	NR	[18]
** Pomalidomide**	0	≤40 %	NR	[21]
(Celgene Corp., Summit, NJ, USA)	11 %	37 %	NR	[22]
	0	17 %	NR	[23]
**JAK Inhibitors**				
** Ruxolitinib (INCB018424)**	44 %	14 %	52–82 %	[42]
(Incyte Corporation, Wilmington, DE, USA—US rights; Novartis AG, Basel, Switzerland—ex-US rights)	42 %^4^	NR	46 %^5^	[43]
	32 %^5^	NR	NR	[44]
** SAR302503 (TG101348)** (Sanofi, Paris, France)	39 %	0	25–89 %	[47]
** CYT387** (Cytopia/YM Biosciences, Mississauga, Ontario, Canada)	45	50 %	NR	[49]
** Pacritinib (SB1518)** (S*Bio Pte Ltd, Singapore)	44 %	<1 %	NR	[50]
** Lestaurtinib (CEP-701)** (Cephalon, Frazer, PA, USA)	18 %	1 %	NR	[51]
**mTOR Inhibitor**				
**Everolimus (RAD001)** (Novartis AG, Basel, Switzerland)	17 %	<1 %	69 %^5^	[52]

IMiDs = immunomodulatory drugs; NR = not reported; NS = not significant.

^1^Percentage of patients achieving ≥50 % reduction in spleen size, unless otherwise specified.

^2^Percentage of patients achieving a ≥2 g/dL increase in Hb, unless otherwise specified.

^3^Percentage of patients achieving complete resolution of symptoms.

^4^Percentage of patients achieving ≥35 % reduction in spleen volume.

^5^Percentage of patients achieving ≥50 % reduction in total symptom score on the Myelofibrosis Symptom Assessment Form.

#### Hydroxyurea (HU)

Despite limited efficacy in reducing spleen size [11,12] and no formal clinical trials to support its use, HU (also known as hydroxycarbamide) remains the most common first-line agent used to treat splenomegaly in MF. In a study evaluating 69 PMF patients [13] treated with HU either alone (55 %) or in combination with erythropoiesis-stimulating agents or anagrelide (45 %), a response to HU as measured by the International Working Group for Myelofibrosis Research and Treatment (IWG-MRT) criteria [14] was documented in 28 % of patients. Multivariate analysis showed a significant association between JAK2V617F positivity and response to HU [13]. Another retrospective study [12] with 40 MF patients treated with HU reported the following symptom improvements: bone pain (100 % of patients), constitutional symptoms (82 %), pruritus (50 %), splenomegaly (40 %), and anemia (12.5 %). Overall clinical improvement (IWG-MRT) was observed in 40 % of patients with a median duration of response of 13.2 months.

In many patients the long-term benefit from HU treatment is curtailed because of progressive loss of efficacy and/or development of resistance or intolerance to HU. Shortcomings of HU treatment include the need for dose titration, which may be limited by cytopenia and a relatively long time (2–3 months) to significant benefit, which is generally not sustained [7].

#### Immunomodulatory drugs (IMiDs)

Thalidomide (Celgene Corp., Summit, NJ, USA), an IMiD with antiangiogenic and anti-inflammatory properties, has shown limited benefit with respect to splenomegaly in MF [15,16]. In one study, only 19 % (4/21) of patients receiving concomitant thalidomide and prednisone therapy achieved splenomegaly reduction ≥ 50 % vs. baseline [15]. In another study, only 8 % (4/50) of patients receiving thalidomide-prednisone alone or in combination with either oral cyclophosphamide or etanercept met the IWG-MRT criteria for clinical improvement of splenomegaly [16].

Lenalidomide (Celgene Corp., Summit, NJ, USA), a second-generation IMiD, is more potent than thalidomide and has a different toxicity profile. In a study of 68 patients with MF treated with lenalidomide, 33 % (14/42) of patients with splenomegaly had a reduction in spleen size by palpation. Lenalidomide also improved anemia in 22 % of patients [17]. The effect of prednisone on the tolerability and response rates of lenalidomide was evaluated in a phase II trial in 40 patients with MF. After a median follow-up of 22 months, the overall response rate was 42 % and 30 % for splenomegaly and anemia, respectively [18]. In a report comparing results with IMiDs in sequential phase II studies conducted at the M.D. Anderson Cancer Center, where MF patients received thalidomide, lenalidomide, or lenalidomide with prednisone, the overall response rate was 16 %, 34 %, and 38 %, respectively (P = 0.06), with a median response duration of 13, 7, and 34 months (P = 0.042), respectively [19]. Although experience is limited, in patients with deletion 5q (del5q; 5q-) chromosomal abnormality, treatment with lenalidomide can result in complete hematologic remission, cause resolution of leukoerythroblastosis, and improve medullary fibrosis. Thus, lenalidomide is recommended as the first-line agent in such patients [20].

Pomalidomide (Celgene Corp., Summit, NJ, USA) is the newest IMiD under investigation for use in MF. In a phase II trial by Tefferi *et al.*, pomalidomide with or without prednisone had no effect on spleen size [21]. In a dose-escalation trial, treatment with pomalidomide resulted in ≥ 50 % reduction in palpable spleen size in 11 % (2/19) of patients [22]. The most recent study evaluated low-dose (0.5 mg/day) pomalidomide in 58 patients with MF. No reduction in splenomegaly was seen [23].

#### Alkylating agents

In a study of low-dose melphalan (2.5 mg 3 times/week with a possible increment to 2.5 mg daily) Petti *et al.*[24] reported a response rate of 66.7 %. Responses in spleen size reduction, classified as complete clinical response (CCR; normalization of spleen size assessed as palpable organ length below the LCM), partial clinical response (PCR; ≥ 50 % reduction in spleen length), and no response (neither CCR nor PCR) were observed in 23 %, 32 %, and 46 % of patients, respectively.

Busulfan has been used to reduce huge splenomegaly and/or severe leuco-thrombocytosis [25]. However, the prolonged administration of busulfan [26] or sequential use of busulfan and hydroxyurea [27] has been associated with an increased incidence of leukemic evolution as well as with other secondary tumors, such as renal cell carcinoma [28].

#### Hypomethylating agents

The hypomethylating agents, 5-azacytidine (Celgene Corp., Summit, NJ, USA) and decitabine (Eisai Inc., Woodcliff Lake, NJ, USA), are currently under investigation for use in MF. In a phase II trial evaluating the efficacy of 5-azacitidine in relapsed/refractory or newly diagnosed MF with poor prognosis, 34 patients received 75 mg/m^2^ 5-azacitidine subcutaneously daily for 7 days every 4 weeks. The overall response rate was 24 %. One patient achieved partial response and 7 had clinical improvement, including 4 of 17 patients with splenomegaly who had a reduction in spleen size [29]. The duration of the response was 4 months. Odenike *et al.* reported the results of a phase II trial with low-dose (0.3 mg/kg/d on days 1–5 and days 8–12) decitabine in patients with MF, in which 7 of 21 patients responded (1 complete remission, 2 partial remissions, and 4 hematologic improvements). The reduction of spleen size was not reported [30].

#### Cladribine (2-chlorodeoxyadenosine; 2-CdA)

Cladribine (Ortho Biotech Products, L.P., Raritan, NJ, USA) has been shown to have some palliative benefit but there is little support for its use in spleen reduction in MF patients. Although one study has reported a response rate (defined as >50 % reduction in liver size, reduction of leukocytosis and thrombocytosis from baseline, and rise of hemoglobin by > 20 g/L) of 64 % after 1–2 treatment cycles, the response was mostly among previously treated, splenectomized (11/14) MF patients. Patients who were not splenectomized (3 patients) had poor response even after more treatment cycles [31].

### JAK2 inhibitors

JAKs are cytoplasmic kinases that play important roles in normal hematopoiesis and proper immune function [32]. Dysregulation of the JAK-STAT pathway is a highly prevalent aberration in patients with MPNs, including MF [33]. A number of alterations, such as excess cytokines and increased JAK1 signaling, as well mutations in JAK2 and mutations involving the thrombopoietin receptor (TPO-r or myeloproliferative leukemia, *MPL*, oncogene) have also been implicated in the etiology and symptomatology of MF, PV, and ET [33-36]. Although JAK2V617F is the most common mutation associated with these MPNs [3,35], it is not necessary for their development [37-39]. Several JAK2 (or JAK1/JAK2) inhibitors are currently in clinical trials for MF. Ruxolitinib (formerly INCB018424; Incyte Corporation, Wilmington, DE, USA) recently became the first FDA-approved drug for the treatment of MF [40] and SAR302503 (formerly TG101348; Sanofi, Paris, France) is in the phase 3 trial for possible approval as therapy for MF. These 2 medications are described here in more detail.

Ruxolitinib is a potent and selective JAK1- and JAK2-inhibitor (IC_50_ of 3.3 and 2.8 nmol/L, respectively, in “naked” kinase assays in cell-free in vitro systems). It demonstrates modest selectivity against Tyk2 (~ 6-fold) and ≥ 130-fold selectivity against JAK3. Treatment with ruxolitinib is associated with a dramatic decrease in circulating levels of proinflammatory cytokines, IL-6, and tumor necrosis factor (TNF)-α, which have been implicated in the pathogenesis of MPNs [41].

The dosing regimen for ruxolitinib was established during a phase I/II trial in 153 patients with primary MF, post-PV MF, or post-ET MF [42]. Sixty-one of 140 patients (44 %) with splenomegaly at baseline had a reduction in spleen size ≥ 50 % by palpation within the first 3 months of therapy. Response was highest among patients receiving 15 mg twice daily (bid; 52 %) and was similar in patients with or without JAK2 mutation. Ruxolitinib-treated patients also demonstrated reductions in spleen volume by MRI. The majority of patients reported a > 50 % improvement in MF-related symptoms. Thrombocytopenia was the dose-limiting adverse event. [42].

Results from 2 randomized phase III trials of ruxolitinib in patients with intermediate-2 or high-risk MF have recently been published. In the Controlled Myelofibrosis Study with Oral JAK Inhibitor Treatment [COMFORT]-I trial MF patients received oral ruxolitinib (n = 155) 15 or 20 mg bid (depending on baseline platelet count) or placebo (n = 154). The primary endpoint, a ≥ 35 % reduction in spleen volume (by MRI or CT) at week 24, was achieved in 41.9 % of ruxolitinib-treated patients vs. 0.7 % of placebo-treated patients (P < 0.001). At week 24, significantly (P < 0.001) more patients in the ruxolitinib group achieved a ≥ 50 % improvement in the Myelofibrosis Symptom Assessment Form (MFSAF) Total Symptom Score (45.9 % vs. 5.3 % for placebo). At the time of a planned safety update with 4 additional months of follow-up, there was a significant survival advantage for ruxolitinib over placebo (hazard ratio = 0.50; P = 0.04). The most common nonhematologic adverse events that occurred more often in the ruxolitinib group were ecchymosis, dizziness, and headache; these were mostly grade 1 or 2. The most common adverse events were hematologic. The rates of grade 3 and 4 anemia and thrombocytopenia in the ruxolitinib group were 45 % and 13 %, respectively, compared to 19 % and 1 %, respectively in the placebo group [43].

In the COMFORT II study patients received ruxolitinib (n = 146) or best available therapy (BAT; n = 73). At week 48, 28.5 % of ruxolitinib-treated patients met the primary endpoint of a ≥ 35 % reduction in spleen volume vs. 0 % in BAT group (P < 0.001). The most common nonhematologic adverse events (all grades) were (ruxolitinib vs. BAT) diarrhea (23 % vs. 12 %) and peripheral edema (22 % vs. 26 %). In the ruxolitinib group, grade 3/4 anemia and thrombocytopenia were reported in 42 % and 8 % of patients, respectively, versus 31 % and 7 % respectively in the BAT group [44]. In both studies, anemia and thrombocytopenia were manageable and rarely led to discontinuation.

SAR302503 is a selective and potent JAK2 inhibitor profiled in 223 kinases and found to have an IC_50_ < 50 nM in 3 kinases— JAK2, FLT3, Ret [45]. It inhibits growth of erythroid colonies in the presence of JAK2V617F, MPL W515K, and JAK2 exon 12 mutations [46] and is 35 and 334 times more selective for JAK2 compared with JAK1 and JAK3, respectively [45]. Pardanani *et al.* recently reported the results of a phase I dose escalation study in which TG101348 was administered in 28-day cycles [47]. The study comprised 59 patients with MF, post-PV MF, or post-ET MF with high/intermediate risk disease and symptomatic splenomegaly unresponsive to available therapy. Many patients with early satiety, night sweats, fatigue, pruritus, and cough at baseline reported rapid and durable improvement in these symptoms. Spleen response was seen within the first 2 cycles of therapy. By 6 and 12 cycles 39 % and 47 % of patients, respectively, had achieved a spleen response (IWG-MRT criteria). No consistent change in plasma cytokine levels was seen, indicating that this agent’s effect on the spleen and the constitutional symptoms may be cytokine-independent. The most common nonhematologic grade 3 or 4 adverse events included nausea (3.4 %), vomiting (3.4 %), and diarrhea (10.2 %). Grade 3 or 4 anemia, neutropenia, and thrombocytopenia was seen in 35.1 %, 10.2 %, and 23.7 % of patients, respectively.

Table 1 summarizes the clinical study findings for these and several other agents currently in clinical trials for MF (some published only in the abstract form).

## Conclusions and future perspectives

MF is a severe, life-threatening, and intensely debilitating disease that has a significant and protracted detrimental effect on patients’ quality of life. Until recently most treatments provided only palliative care with no single treatment addressing all of the complications and symptoms of the disorder. Although allogeneic stem cell transplant offers the potential for cure, it is associated with a high mortality rate, even using a reduced intensity protocol, and thus is only appropriate for a limited group of patients (e.g., younger, otherwise healthy patients with high-risk MF). The discovery of a JAK2 mutation (JAK2V617F) and the dysregulated JAK-STAT activity that is common in patients with MF, PV, and ET has led to the investigation of several agents that focus on inhibition of JAK enzymatic activity. Clinical study results to date indicate that the primary therapeutic benefits of these therapies are a reduction in splenomegaly and significant improvement in MF-related symptoms. These improvements are generally seen within 1 to 2 months of initiating therapy and appear to be durable. The adverse event profiles of the JAK inhibitors vary, but the most common clinically significant adverse effect is dose-related myelosuppression. As yet, no significant, durable improvement in bone marrow fibrosis has been reported with any of the therapies, and the effect of JAK inhibitors and other novel agents under development on the JAK2V617F allelic burden has been inconsistent. Since no JAK2 inhibitor in clinical development so far have been shown to be selective for JAK2V617F mutation, and at the enzymatic level they inhibit both mutated and wild-type JAK2 enzyme, it is not surprising that the elimination of a JAK2 mutated clone in patients on therapy has not been seen. Rather, JAK2 inhibitors work equally well for MF patients with or without JAK2 mutation. It is plosible that mode of action of JAK2 inhibitors is primarily anti-proliferative since JAK2 is involved in essential hematopoiesis. Those JAK2 inhibitors that also inhibit JAK1 (e.g. ruxolitinib) appear to provide also anti-inflammatory effect, evidenced by the significant reduction of inflammatory and other cytokines in blood of patients on therapy. There remain a number of unanswered questions concerning the exact mechanism of action of these agents.

Although the JAK inhibitors are not curative, the reductions in splenomegaly and systemic symptoms are clinically significant and important to patients. Further clinical development of ruxolitinib as well as the other JAK inhibitors can be expected. Optimal timing of treatment, dosage, and duration of therapy will become better defined. For example, the use of JAK inhibitors as first line therapy in patients with mild splenomegaly to prevent spleen progression has to be investigated since clinical experience with these agents so far has been limited to patients with advanced MF and symptomatic very enlarged spleen. Evaluation of the use of JAK inhibitors in specific clinical situations is warranted, for example, the use of JAK inhibitors in pre-bone marrow transplant (BMT) period to allow for BMT in better patients’ condition is mandatory as BMT stays the only way to eliminate the disease and potentially cure the patients. Similarly, their use in patients with splenomegaly due to splanchnic vein thrombosis in which the mechanism of splenomegaly is probably different from that of typical myelofibrosis is warranted as well. Furthermore, combination therapy, likely based on a ruxolitinib backbone, may further improve outcomes.

Despite the obvious need for additional research, available data indicate that JAK inhibitors are poised to provide meaningful long-term benefits (possibly including improved survival as seen in the COMFORT-I trial) to patients with this serious, chronic, debilitating, and potentially lethal disease. Currently we advocate the use of ruxolitinib as the first option for the patients with symptomatic, significant splenomegaly, that have intermediate- and high-risk PMF or secondary MF. There are select cases, however, where splenomegaly can (and should) be controlled by either splenectomy or splenic irradiation.

## Competing interests

EA has received research support from Incyte. SV has received research support from Incyte Corporation, Cephalon, Bristol Myers Squibb, AstraZeneca, Celgene, Geron, Gilead, Infinity, Roche, Lilly, YM Bioscience, NS Pharma, SBio, and Exelixis for a conduct of clinical studies.

## Authors’ contributions

All authors have contributed in writing the manuscript. The authors take full responsibility for the content of this article. The authors did not receive financial compensation for authoring or publishing the article. All authors have read and approved the final manuscript.
